# Verbesserte Patientenversorgung und effizientere Zusammenarbeit zwischen Apotheken und Pflegediensten durch maschinelle Medikamentenverblisterung und die Vernetzungsplattform MediMan: Eine Fallstudie

**DOI:** 10.1365/s40702-022-00879-4

**Published:** 2022-05-27

**Authors:** Patricia Kajüter, Alina Behne, Frank Teuteberg

**Affiliations:** Fachgebiet „Unternehmensrechnung und Wirtschaftsinformatik“, Universität Osnabrück, Osnabrück, Deutschland

**Keywords:** Verblisterung, Vernetzungsplattformen, Prozessverbesserung, Medikationsmanagement, Patientenversorgung, Blistering, Digital linking platforms, Process improvement, Medication management, Patient care

## Abstract

Pflegebedürftige können ihre Medikamente oft nicht selbstständig stellen, deshalb wird dies durch geschultes Fachpersonal ambulanter Pflegedienste übernommen. Dieser Prozess ist fehleranfällig, denn die Medikamente werden häufig manuell und in der Nachtschicht vom Pflegefachpersonal zusammengestellt. Hierdurch kann es zu Risiken wie Doppelmedikationen und unberücksichtigten Wechselwirkungen unterschiedlicher Medikamente kommen. Zudem ist dieser Prozess nicht ressourceneffizient und durch den bereits bestehenden Fachkräftemangel nicht tragbar. Durch die vergleichsweise geringe digitale Vernetzung im deutschen Gesundheitswesen gibt es bei der interprofessionellen Zusammenarbeit zwischen Apotheken, Pflegediensten, Blisterzentren und Arztpraxen weiteren Verbesserungsbedarf. Ziel dieses Beitrags ist es daher aufzuzeigen, welche Erkenntnisse und Handlungsbereiche sich aus einer Fallstudie zur digitalen Vernetzungsplattform MediMan ableiten lassen und wie in diesem Zusammenhang die maschinelle Medikamentenverblisterung dazu beitragen kann, die interprofessionelle Zusammenarbeit zwischen Apotheken, Pflegediensten, Blisterzentren und Arztpraxen durch eine sichere und effiziente Patientenversorgung zu führen. Anhand einer Prozessmodellierung, basierend auf Experteninterviews und Fokusgruppen mit Stakeholdern aus dem Gesundheitswesen, wird aufgezeigt, welche Potenziale sich aus der maschinellen Verblisterung in Kombination mit einer Vernetzungsplattform ergeben. Als Ergebnis lassen sich acht Erkenntnisse bzw. Handlungsbereiche ableiten, die in diesem Beitrag vertiefend vorgestellt werden: (1) Prozessvereinfachung, (2) Plattformerprobung, (3) Abstimmungsbedarfe, (4) Mitarbeitereinbindung und -schulung, (5) Informationsverbreitung, (6) Vergütungsregelungen, (7) Schnittstelle Warenwirtschaftssystem sowie (8) Interoperabilität und Telematikinfrastruktur.

## Abstract (en)

People in need of care often cannot put together their own medication, so this is done by trained specialist staff from outpatient care services. This process is prone to errors, because the medications are often put together manually by the nursing staff during the night shift. This can lead to risks such as double medication and unconsidered interactions of different medications. In addition, this process is not resource-efficient and is not sustainable due to the already existing shortage of skilled personnel. Due to the comparatively low level of digital linking in the German healthcare system, there is further need for improvement in interprofessional collaboration between pharmacies, nursing services, blister centers and doctors’ practices. The aim of this paper is therefore to show which findings and areas of action can be derived from a case study on the digital linking platform MediMan and how, in this context, machine-based medication blistering can help to guide interprofessional collaboration between pharmacies, nursing services, blister centers and doctors’ practices through safe and efficient patient care. A process model based on expert interviews and focus groups with stakeholders from the healthcare sector is used to show the potentials that arise from automated blistering in combination with a networking platform. As a result, eight findings and areas for action can be derived, which are presented in more detail in this article: (1) Process simplification, (2) platform testing, (3) coordination requirements, (4) employee integration and training, (5) information dissemination, (6) remuneration regulations, (7) interface to the enterprise resource planning system, as well as (8) interoperability and telematics infrastructure.

## Relevanz der maschinellen Verblisterung für die Pflege

Im Dezember 2019 lag die Anzahl pflegebedürftiger Menschen in Deutschland bei 4,13 Mio., was in etwa fünf Prozent der Bevölkerung entspricht, Tendenz steigend (Destatis 2021a). Die Zahl der Pflegebedürftigen hat sich dabei seit 2009 beinahe verdoppelt. Die Überalterung der Gesellschaft als Folge soziodemografischer Entwicklungen zeichnet sich dadurch aus, dass ein überproportionaler Anstieg von Bürgern mit medizinischem und pflegerischem Versorgungsbedarf vorliegt. Insbesondere die ältere Bevölkerung leidet häufiger an chronischen Krankheiten im Vergleich zum jüngeren Teil der Bevölkerung und ist oftmals multimorbid (Laurenza et al. 2018). Die Anzahl an Senioren wird voraussichtlich von 15,9 Mio. in 2018 auf mindestens 21 Mio. in 2039 wachsen (Destatis 2021b). Zugleich herrscht im Gesundheitswesen und insbesondere im Bereich der Pflege ein Fachkräftemangel, welches die Versorgungssituation zusätzlich verschärft und den erheblichen Bedarf nach Möglichkeiten der Zeiteinsparung im Arbeitsalltag von Pflegekräften verdeutlicht (Seyda et al. 2021).

Eine der größten Herausforderungen für die ältere Bevölkerung mit chronischen und schweren Krankheiten wie z. B. Demenz liegt darin, ihren vom Arzt verschriebenen Medikationsplan zu lesen und einzuhalten (Laurenza et al. 2018). Um Medikationsfehlern wie der falschen Einnahme oder dem Vergessen der Einnahme von Seiten der Patienten vorzubeugen, können pflegende Angehörige unterstützen oder Pflegedienste beauftragt werden, die Medikation für den Patienten zusammenzustellen und zu verabreichen. Die Medikamentenversorgung durch Pflegedienste ist jedoch ein Hochrisikoprozess und kann hohe Fehlerquoten aufweisen, denn die Medikamente werden häufig manuell vom Pflegepersonal und meist in der Nachtschicht zusammengestellt (Lauterbach et al. 2007; KU 2020; Negele und Königer 2019). Uhrhan und Schaefer (2010) zufolge ist das Stellen der Medikationen in der Nachtschicht als besonders bedenklich anzusehen, da solch ein fehleranfälliger und zeitaufwändiger Prozess in störungsfreien und gut belichteten Räumen durchgeführt werden sollte, welches in Nachtschichten häufig nicht gegeben ist. Van den Bemt et al. (2009) fanden in einer Studie zur Medikationsverabreichung in Pflegeheimen heraus, dass die häufigsten Fehler bei der Verabreichung der Medikation im Rahmen der Zusammenstellung der Medikationen durch das Pflegepersonal passieren. Insgesamt waren 21 % der gestellten Medikationen fehlerhaft. Zu einem ähnlichen Ergebnis kommen Bader et al. (2003), welche postulieren, dass in ihrer Studie bei 28 % der Bewohner von Pflegeheimen Fehler bei der Stellung von Medikationen aufgetreten sind. Die manuelle Sortierung und Verabreichung der Medikamente für den Patienten bergen Risiken wie Doppelmedikation und nicht berücksichtigte Wechselwirkungen unterschiedlicher Medikamente (Negele und Königer 2019). Eine mögliche Lösung, um dem entgegenzuwirken, könnte die Unterstützung des Pflegepersonals oder der pflegenden Angehörigen durch eine Apotheke mittels der maschinellen Verblisterung von Medikamenten über Blisterzentren darstellen. Darüber hinaus können die Risiken der Medikationsfehler durch uneindeutige Dokumentationen ärztlicher Anweisungen, Kommunikationsprobleme und unstrukturierter Aufbewahrung der Medikationen weiter erhöht werden (Uhrhan und Schaefer 2010), welches den Bedarf nach verbesserter Vernetzung der Stakeholder im Gesundheitswesen verdeutlicht.

Im Rahmen dieses Artikels wurde eine Fallstudie mit einer Apotheke, einem Pflegedienst und einem Blisterzentrum durchgeführt, in der elf ursprünglich nur vom Pflegedienst manuell versorgte Patienten durch maschinelle Verblisterung und unter Anwendung der Vernetzungsplattform MediMan in ihren Wohngemeinschaften neuartig versorgt wurden. Das Ziel des Artikels ist es daher, Erkenntnisse und Handlungsempfehlungen aufzuzeigen, wie eine digitale, über die Apotheke gesteuerte Vernetzungsplattform MediMan im Zusammenspiel mit der maschinellen Medikamentenverblisterung zu einer sicheren und effizienten Patientenversorgung sowie einer verbesserten interprofessionellen Zusammenarbeit zwischen Apotheken, Pflegediensten, Blisterzentren und Arztpraxen führen kann. In diesem Zuge wird ein Prozess zur maschinellen Medikamentenverblisterung dargestellt. Es werden daher die folgenden Forschungsfragen verfolgt:

### FF1

Welche Potenziale ergeben sich aus der maschinellen Medikamentenverblisterung kombiniert mit einer Vernetzungsplattform, um die Zusammenarbeit zwischen Apotheken und Pflegediensten zu verbessern?

### FF2

Welche Erkenntnisse und Handlungsbereiche lassen ich aus der vorliegenden Fallstudie in Bezug auf die maschinelle Verblisterung, gesteuert über die Vernetzungsplattform MediMan ableiten?

Im Folgenden wird zuerst ein Überblick über die Grundlagen der Verblisterung gegeben, indem relevante Begrifflichkeiten definiert, der Status Quo der Verblisterung beschrieben sowie die Vernetzungsplattform MediMan dargestellt wird. Daran anknüpfend werden die rechtlichen Rahmenbedingungen für die Verblisterung erläutert. Anschließend wird die zugrundeliegende Methodik dieser Fallstudie dargelegt. Es werden daraufhin der Ausgangsprozess der Verblisterung erläutert, Schwachstellen identifiziert und darauf aufbauend ein Zielprozess sowie Potenziale der Plattform MediMan analysiert. Die Ergebnisse werden kritisch diskutiert, Implikationen und Limitationen werden aufgezeigt und ein Ausblick für zukünftige Forschung gegeben. Abschließend werden die Ergebnisse in einem Fazit mit Rückblick auf die Forschungsfragen zusammengefasst.

## Grundlagen der Verblisterung

### Definitionen

Bei der Verblisterung werden verordnete Medikamente eines Patienten nach spezifischen Einnahmezeitpunkten (Wochentagen und Tageszeiten) portioniert und in durchsichtige, beschriftete Einmaltüten (Blister) verpackt. Für die Verblisterung werden diese Arzneimittel aus den ursprünglichen Verpackungen genommen und je nach Medikation der Patienten in einer Apotheke zusammengestellt und neu verpackt. Dieser Prozess der Verblisterung kann sowohl manuell als auch maschinell erfolgen. Der Zeitraum der Versorgung entspricht dabei in der Regel einer Woche. Hierbei handelt es sich in den meisten Fällen um feste orale Darreichungsformen in Form von Tabletten, Kapseln oder Dragees (IQWiG 2019; ZLG 2010).

**Abb. 1:**
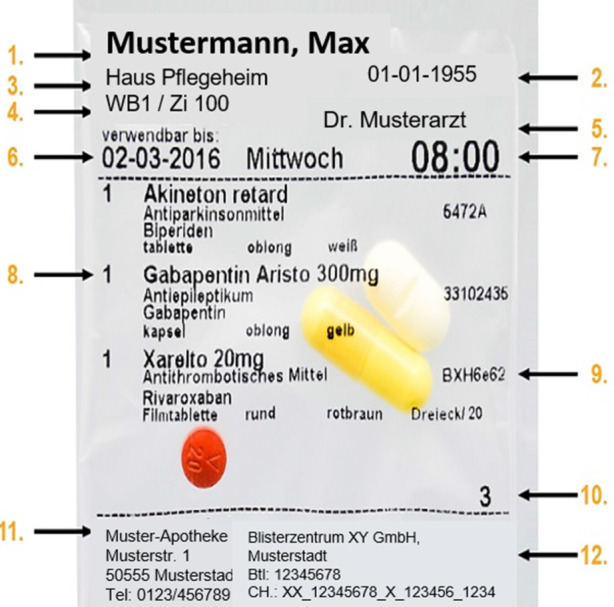
Beispiel für einen Schlauchblister. (1. Name des Patienten, 2. Geburtsdatum, 3. Name der Pflegeeinrichtung, 4. Wohnbereich, 5. Name des Arztes, 6. Verfallsdatum, 7. Einnahmezeitpunkt, 8. Arzneimittelname (Menge & Indikation, Wirkstoff & Beschreibung), 9. Chargenbezeichnung, 10. Anzahl Tabletten pro Schlauchblister, 11. Name der Apotheke, 12. Herstellerinformationen)

Als Teil der Verblisterung spielen sogenannte Schlauchblister eine Rolle. Bei diesen Schlauchblistern werden die Arzneimittel, die für den jeweils gleichen Einnahmezeitpunkt verblistert werden sollen, in einer durchsichtigen Folientüte eingeschweißt. Auf diese Folientüte wird der Name des Patienten, Einnahmehinweise, Informationen zu den Arzneimitteln sowie das Verfallsdatum aufgedruckt (IQWiG 2019). Diese Vorgänge lassen sich auch maschinell über Blisterautomaten, insbesondere in Blisterzentren, durchführen. Blisterzentren können sowohl als Herstellbetriebe agieren, die für „fremde“ Apotheken verblistern, als auch direkt von Apotheken selbst betrieben werden. Ein Beispiel für einen Schlauchblister lässt sich Abb. 1 entnehmen.

### Status Quo der Verblisterung

Bei der Zusammenstellung der Medikation hinsichtlich individueller Einnahmezeitpunkte kann es vorkommen, dass Änderungen der Medikation der Patienten, die auf ausgestellten Rezepten auf Papier stehen, verloren gehen. Deshalb ist ein regelmäßiger Austausch zwischen Apotheke und Pflegedienst von Relevanz und jede Änderung der Medikation zwingend zu dokumentieren. Bei der Kooperation mit einer Apotheke bedeutet dies einen erhöhten Abstimmungsbedarf für Pflegedienste, weil die neuen Blister auf die Medikationsänderung anzupassen sind. Darüber hinaus besteht bei neuer verschreibungspflichtiger Medikation durch Einholung eines neuen Rezepts beim Arzt ein erhöhter logistischer Aufwand der Apotheke (Springer Medizin 2016). Aktuell laufen die Abstimmungen zwischen Pflegedienst, Arztpraxis und Apotheke in der Regel über Fax und Telefon. Die Anpassungen der Verblisterung (Ändern und Nachliefern) wird von der Apotheke gesteuert. Bei der manuellen Verblisterung besteht das Risiko von Fehl- oder Doppelmedikationen, wenn Medikamente falsch zusammengestellt werden. Möhlmann und Traupe (2011) postulieren deshalb, dass der Einsatz der maschinellen Verblisterung Fehl- und Doppelmedikationen deutlich reduzieren kann und dadurch Krankenhauseinweisungen gesenkt werden können, was zu Kosteneinsparungen sowie einer höheren Patientensicherheit führen kann. Da bei der maschinellen Verblisterung auf jeden Schlauchblister sämtliche relevante Informationen des Patienten, der Arzneimittel sowie der Einnahme abgedruckt werden, erhalten Apotheker, Pflegedienste und Patienten so die Möglichkeit, bei Bedarf jederzeit die Medikation zu überprüfen und so mehr Bezug zu den gegebenen Arzneimitteln zu erhalten.

Bereits heute bestehen technologisch unterstützte Ansätze im Rahmen der Verblisterung. Es gibt einfache Lösungen wie eine 7‑Tage-Pillendose mit Alarmfunktion oder umfassendere Lösungen zum Medikationsmanagement zuhause wie das Dosier- und Kommunikationssystem Philips Medido oder maja sana (2021). Solche Lösungen können die verblisterten Medikamente für den Patienten in der richtigen Dosierung ausgeben und zusätzlich mit einer Gesundheitsplattform verbunden sein. Anhand des von der Apotheke in das System eingetragenen Medikationsplans unterstützt der automatische Tablettendispenser den Patienten bei der rechtzeitigen und korrekten Einnahme der verordneten Medikamente. Mithilfe dieser Lösungen können potenzielle Fehl- oder Doppelmedikationen reduziert werden und ein längeres, selbstbestimmtes Leben in den eigenen Wohnräumen ermöglicht werden (maja sana 2021). Neben der Vernetzung von Apothekern und Patienten bestehen auch Plattformen zwischen Apotheker und Blisterzentrum, um die Abwicklung der maschinellen Verblisterung zu erleichtern. Das aktuelle Standardprogramm von Blisterzentren ist Blister.connect von Pharmatechnik, das einen Austausch auf Datenebene ermöglicht. Dadurch können Aufträge hochgeladen, Rückmeldungen und Alternativen zu nicht verfügbaren Medikamenten angezeigt sowie ganze Bestellungen übersichtlich eingesehen werden^1^.

### Vernetzungsplattform MediMan

Die bereits bestehenden Lösungen der technologisch unterstützten Ansätze im Rahmen der maschinellen Verblisterung sind Insellösungen, die sich meist nur auf die Kommunikation zwischen zwei Akteursgruppen beschränken. Eine einheitliche Lösung, bei der über eine Schnittstelle direkt aus dem Medikationsplan im Medikationsmanagementsystem eine Bestellung ausgelöst werden kann und die Kommunikation verschiedener Akteure auftragsorientiert über einen Chat ermöglicht, bietet die Vernetzungsplattform MediMan^2^. Ein solcher Lösungsansatz wurde im Rahmen dieser Fallstudie zum Einsatz dieser Plattform untersucht. Dieser Ansatz umfasst eine bereichsübergreifend einheitliche Patientendokumentation, ermöglicht ein automatisches Rezeptmanagement mittels Reichweitenberechnung, sowie Medikamentenpläne und soll zukünftig auch eine Schnittstelle zu Warenwirtschaftssystemen bieten. Patienten als Apothekenkunden können die Plattform nutzen, indem sie Logins, d. h. Anmelde- bzw. Zugangsdaten, von der Apotheke erhalten. So können sie schließlich in der Anwendung selbst verschiedene Funktionen wie die Datenverwaltung, die Kontaktaufnahme zur Apotheke per Chat oder die Organisation und Bestellung der Arzneimittel nutzen. Für die Zusammenarbeit der Apotheke mit anderen Gesundheitsdienstleistern wie Pflegedienst, Blisterzentrum oder Arztpraxis ist die Befugnis der Patienten notwendig. Bei Pflegebedürftigen können Pflegedienst und Apotheke – in Absprache mit der zuständigen Arztpraxis – das Medikationsmanagement übernehmen. Diese Plattform dient als Schnittstelle, die eine digitale Kommunikation zwischen den Gesundheitsakteuren ermöglicht und dokumentiert. Auf die Medikationsmanagementsoftware könnten diverse Akteure des Gesundheitswesens zugreifen, wobei in dieser Fallstudie der Fall beschrieben wird, dass ein Pflegedienst und die Apotheke aktive Nutzer der Plattform MediMan sind. Die Angaben der Nutzer werden verschlüsselt und jegliche Daten- und Kommunikationswege nach den Vorgaben der Datenschutz-Grundverordnung (DSGVO) geschützt.

### Rechtliche Rahmenbedingungen

Als Grundlage für das Stellen und Verabreichen von Medikamenten gilt SGB V § 37, welches die medizinische Behandlungspflege beschreibt, die jegliche Tätigkeiten, die auf Verordnung von Ärzten hin von Pflegekräften durchgeführt werden, umfasst. Dies beinhaltet u. a. die Medikamentengabe. Hausärzte können sowohl ein Rezept zum Stellen als auch zur Verabreichung ärztlich verordneter Medikamente ausstellen, welche von der Krankenkasse abgerechnet werden. Diese können auch in Kombination ausgestellt werden. Hierbei ist zu beachten, dass ein solches Rezept nur dann ausgestellt wird, wenn der Patient nicht in der Lage ist, Medikamente alleine oder mit Hilfe von im Haushalt lebenden Angehörigen einzunehmen, weil beispielsweise kognitive Erkrankungen oder Bewegungseinschränkungen vorliegen. In diesem Fall wird dies durch den Pflegedienst übernommen (VdK 2021).

Es gibt einige rechtliche Rahmenbedingungen und Anforderungen, die zu erfüllen sind, um in Deutschland sowohl die manuelle als auch die maschinelle Verblisterung durchführen zu dürfen. Das Verblistern gilt als Herstellung von Medikamenten. Rechtlich wird das Verblistern mit dem Herstellen einer Rezeptur gleichgesetzt. Die Apotheke vor Ort besitzt bereits durch die Apothekenbetriebsordnung die Erlaubnis, Rezepturen anzufertigen. Die Verblisterung ist ein Herstellungsprozess, da das Verpacken von Medikamenten eine Veränderung darstellt. Ein Blisterzentrum bekommt die notwendige Herstellungserlaubnis ausgestellt, wenn Qualitätsansprüche im Rahmen einer Guten Herstellungspraxis (Good Manufacturing Practice) gewährleistet werden können (GMP-Verlag 2020). Ist dies gegeben, schließen Blisterzentrum und Apotheke einen schriftlichen Vertrag, in dem Verantwortlichkeiten festgehalten sind. Die Apotheke steht auch bei Beauftragung eines Blisterzentrums in der Verantwortung für die Richtigkeit und Qualität der verblisterten Medikamente, da die Apotheke die letzte Instanz ist, die Arzneimittel ausgeben darf (Saalfrank 2014).

Mit dem Beschluss des Digitale-Versorgung-und-Pflege-Modernisierungs-Gesetz in 2021 eröffnen sich neue Potenziale für die Digitalisierung des deutschen Gesundheitswesens. Dies könnte auch für die maschinelle Verblisterung von Relevanz sein, weil neben der Zeiteinsparung die automatische Übertragung des Medikationsplans in maschinell hergestellte Blister auch weniger Übertragungsfehler aufweist als das manuelle Stellen (BPAV 2021). Insbesondere der Ausbau der Telematikinfrastruktur (TI), in unserem Anwendungsfall die Einführung des E‑Rezeptes, des Kommunikationsdienstes KIM sowie des elektronischen Medikationsplans, erleichtert die Möglichkeiten zur Ausweitung des Angebots, da digitale Lösungen die Kommunikation zwischen und zu den Gesundheitsdienstleistern vereinfachen können und automatisierte Lösungen potenziell mehr Aufschwung erhalten können (Klein und Schellhammer 2017). Apotheken mit mindestens 4,65 Mio. abgegebenen Packungen pro Jahr fallen zudem unter die Kritischen Infrastrukturen Deutschlands (KritIs), welche durch die Verordnung zur Bestimmung Kritischer Infrastrukturen nach dem BSI-Gesetz (BSI-Kritisverordnung – BSI-KritisV) definiert werden. Sie werden somit als besonders schützenswürdig angesehen. Neben der BSI-KritisV greift auch das IT-Sicherheitsgesetz, welches zum Ziel hat, die IT-Systeme, Komponenten und Prozesse Kritischer Infrastrukturen zu schützen. Hierbei werden KritIs-Betreiber dazu verpflichtet, bestimmte Anforderungen zu erfüllen, wie die Benennung einer Kontaktstelle, der Meldung von IT-Störungen und Sicherheitsvorfällen, der Umsetzung des aktuellen Stands der Technik, der Ausarbeitung von Präventionsmaßnahmen und Reaktionsplänen sowie der Prüfung der Absicherung wie z. B. durch Sicherheitsaudits (TÜV Nord 2021).

## Methodik

In diesem Beitrag wird anhand einer Prozessdarstellung aufgezeigt, welche Herausforderungen die manuelle Rezept- und Medikamentenbestellung und das manuelle Verblistern durch den Pflegedienst darstellt sowie welche Potenziale sich durch die maschinelle Verblisterung und den Einsatz einer Vernetzungsplattform ergeben. Die Prozessdarstellung folgt der Prozessmodellierungssprache Business Process Model and Notation, wobei diese in Anlehnung an Wohed et al. (2006) in Teilen vereinfacht verwendet wird, um den relativ komplexen Anwendungsfall verständlicher darzustellen. Die zugrundeliegende Fallstudie fand im Bereich des Prozessmanagements statt, um den Ablauf und die Kommunikation bei der Medikamentenbestellung, -lieferung sowie -vergabe durch Pflegedienste mithilfe digitaler Technologien zu verbessern und so das Patientenwohl und die Patientensicherheit zu erhöhen. Dieses Vorhaben orientiert sich am Geschäftsprozessmanagement-Lebenszyklus, bei dem sukzessiv die folgenden fünf Schritte durchgeführt werden: Prozesserhebung, Prozessmodellierung, Prozessanalyse, Prozessverbesserung und Prozessimplementierung (Dumas et al. 2013). Die Erprobung der Vernetzungsplattform MediMan, die mithilfe einer wissenschaftlichen Literaturrecherche, Workshops und Fokusgruppen (Morgan 1997), drei Experteninterviews mit Apothekern von an der Fallstudie beteiligten Apotheken sowie Prozessanalysen (Gläser und Laudel 2010) begleitet wurde, dient als Basis dieser Arbeit. Es fanden insgesamt zwei Workshops mit jeweils vier bzw. sechs potenziellen Nutzern von MediMan sowie zwei Fokusgruppen mit fünf Experten, bestehend aus Apothekern, Mitarbeitern in Apotheken, Pflegedienstleitungen und Mitarbeitern von Pflegediensten, die an dieser Fallstudie teilgenommen haben, statt.

## Ausgangssituation und Zielprozess

### Ausgangssituation und Schwachstellenidentifikation

**Abb. 2:**
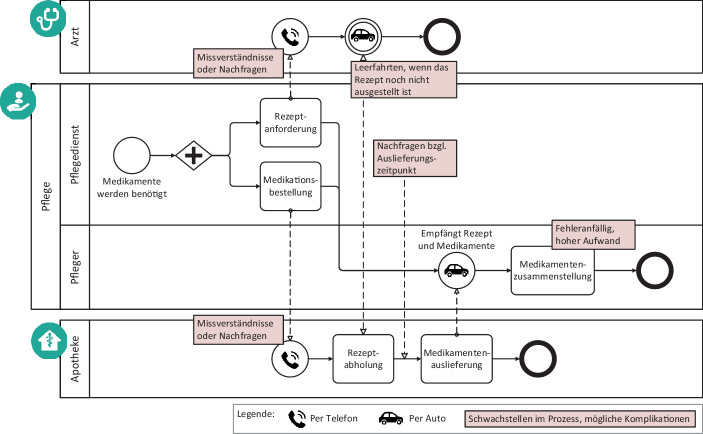
Prozessdarstellung der aktuellen Versorgung pflegebedürftiger Patienten über den Pflegedienst

Die Medikationsversorgung pflegebedürftiger Personen erfolgt aktuell mithilfe eines Medikationsplans, anhand dessen die Medikation bei den Pflegediensten für den Patienten manuell zusammengestellt wird. Dieser Medikationsplan liegt entweder ausgedruckt vor oder digital in einem System, auf das in der Regel jeweils nur eine Institution Zugriff hat. In vielen Pflegediensten stellt der manuelle Prozess – vom Anrufen bei der Arztpraxis und Apotheke bis hin zum Stellen der Medikation – einen hohen Aufwand für das bereits überlastete Personal dar. Die Kommunikation zwischen den Patienten, Apotheken und Arztpraxen, um Rezepte und Medikation zu bestellen, erfolgt in der Regel telefonisch oder per Fax. Dies kann Missverständnisse, lange Wartezeiten, Leerfahrten und Fehler begünstigen. Abb. 2 verdeutlicht die Schritte, die notwendig sind, bis ein Patient über einen Pflegedienst seine Medikation erhält.

Im Rahmen der Fokusgruppen wurden die Erwartungen der einzelnen Akteursgruppen für einen einfachen Zielprozess bestimmt. Dafür wurde der Prozess der manuellen Verblisterung als Basis genommen und erfragt, welche Potenziale sich in dem vernetzten Zielprozess ergeben können. Der Zielprozess orientierte sich an den bisherigen Möglichkeiten der Vernetzungsplattform MediMan, die in der Apotheke vor Ort zum Einsatz kommen kann. Mithilfe der Vernetzungsplattform sollen die im Voraus identifizierten Probleme hinsichtlich der Kommunikation und einer fehlenden Datenbasis gelöst werden. Tab. 1 präsentiert die Erwartungshaltung der befragten Apotheker und Mitarbeiter der Pflegedienste an den durch maschinelle Verblisterung und die Vernetzungsplattform MediMan unterstützten Zielprozess.

**Tab. 1:** Erwartungshaltung an den Zielprozess

Akteursgruppe	Bestellung	Auslieferung	Lebensqualität und Sicherheit	Ziele für den Arbeitsalltag
Patient/in	Wenig Aufwand, kaum Abstimmung	Kontinuierliche Versorgung auch bei Engpässen	Höhere Lebensqualität	Weniger Aufwand für hohe Lebensqualität
Pflegedienst	Standardisierte Bestellung ohne Warteschleifen	Planungssicherheit	Sichere Versorgung von Patienten	Entlastung, mehr Zeit für die Pflege
Blisterzentrum	Automatisiert weiter zu verarbeitendes Datenformat	Bestimmter Ansprechpartner	Einfache Anwendung bei Vergabe	Absatzsteigerung
Arztpraxis	Wenig Kommunikation	Kein/kaum Aufwand	Weniger Komplikationen	Entlastung
Apotheke	Automatisiert aus Medikationsplan	Entlastung durch fertig geblisterte Lieferung	Höhere Arzneimitteltherapiesicherheit	Kundenbindung, klare Verantwortlichkeiten, Absatzsteigerung

### Zielprozess und Potenziale der Plattform MediMan

**Abb. 3:**
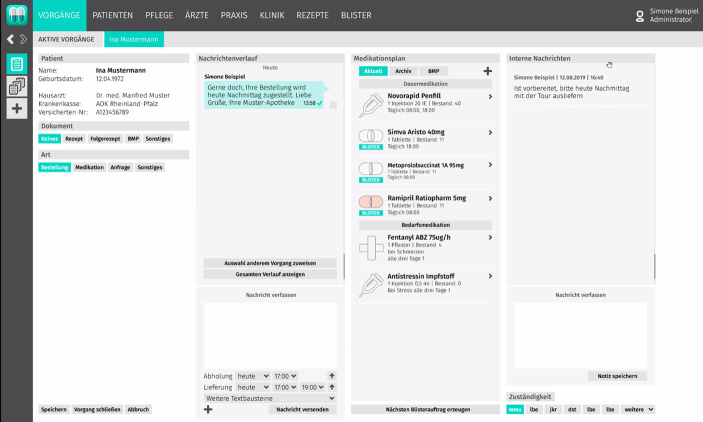
Graphische Benutzerschnittstelle (GUI) von MediMan

Das Ziel ist es, einen standardisierten und teilautomatisierten Prozess mithilfe einer Plattform – in diesem Fall MediMan – zur maschinellen Medikamentenverblisterung zu erreichen. Die graphische Benutzerschnittstelle der MediMan-Plattform lässt sich Abb. 3 entnehmen.

**Abb. 4:**
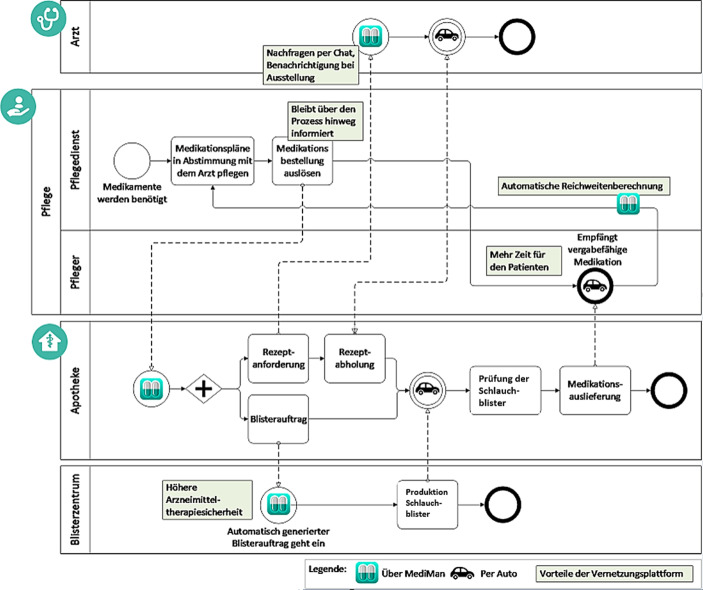
Prozessdarstellung der Patientenversorgung durch maschinelle Verblisterung unterstützt durch die Vernetzungsplattform MediMan

Der Zielprozess sieht wie folgt aus: Im Vorhinein einigen sich Pflegedienst und Apotheke über den zeitlichen Ablauf der Medikationsbestellung und -lieferung. Der Zeitraum zwischen Rezeptanfrage und Medikationslieferung beläuft sich auf vier bis fünf Tage. Auf der Plattform kann die Institution ihre Nutzer und Abteilungen sowie die Patienten mit Zugriffsberechtigungen individuell einrichten. Der Pflegedienst hat die Möglichkeit jederzeit, insbesondere nach Abstimmung mit dem zuständigen Arzt, auf der Plattform MediMan den Medikationsplan einzurichten und zu pflegen. Die Apotheke kann dabei unterstützen und den Medikationsplan einsehen, die Verträglichkeit der Medikation prüfen und Änderungen am Medikationsplan vornehmen. Apotheke und Pflegedienst arbeiten im Team in Abstimmung mit dem zuständigen Arzt gemeinsam an Medikationsplänen, um die Synergieeffekte zu nutzen. Zu jedem Zeitpunkt werden Änderungen im System anhand des verwendeten Logins dokumentiert. Zum vereinbarten Zeitpunkt löst der Pflegedienst Rezeptbestellungen für Bedarfsmedikation per Knopfdruck aus. Daraufhin erhält die Apotheke einen Auftrag im Bereich „Rezeptanforderungen“ in MediMan. Wird dieser bestätigt, erhält die zuständige Arztpraxis die Rezeptanfrage entweder gewohnt per Fax oder, falls Bereitschaft besteht, auch über einen eigenen Arztpraxis-Login digital. Rezeptbestellungen für einen Blisterauftrag werden meist automatisch generiert, wobei zurzeit noch eine manuelle Ausführung durch die Apotheke vor Ort erfolgt bis in MediMan implementiert ist, die Reichweitenberechnung und folgerichtig die Folgerezeptbestellung automatisch auszulösen. Dieser Prozess ist in Abb. 4 graphisch dargestellt.

**Abb. 5:**
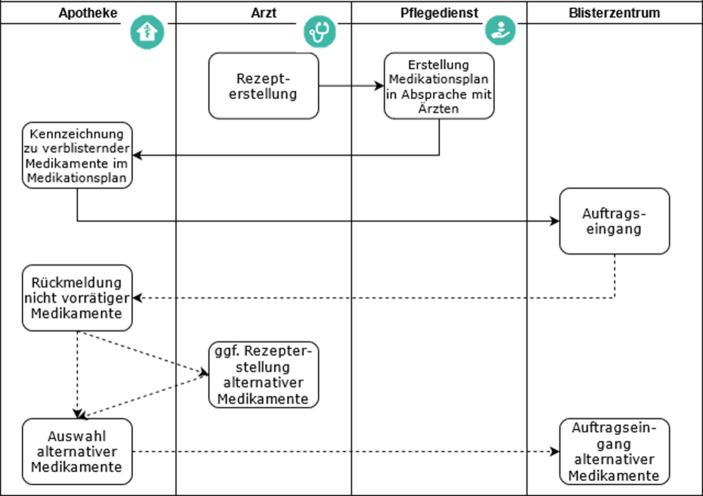
Datenflussdiagramm zum Auftragseingang zur maschinellen Verblisterung über MediMan

Damit das Zusammenspiel zwischen Apotheke und Blisterzentrum automatisiert über die Plattform MediMan laufen kann, ist eine Schnittstelle zur Blistersoftware herzustellen. Für die Abrechnung ist das Rezept derzeit noch manuell in das Warenwirtschaftssystem hochzuladen. Nichtsdestotrotz könnte der strukturierte Prozess mit der neuen Vernetzungsplattform bereits jetzt den Arbeitsaufwand erleichtern, da MediMan viele Insellösungen integriert und nur noch das Warenwirtschaftssystem daneben einzusetzen ist. Für die Schnittstelle von MediMan zum Blisterzentrum ist das entsprechende Datenformat von Bedeutung, aus dem ein Schlauchblister erzeugt werden kann. Im Allgemeinen kann die Apotheke die Medikamente in den Medikationsplänen kennzeichnen, die verblistert werden sollen. So muss die Apotheke nur noch die vom Pflegedienst vorgeschlagenen Patienten auswählen und die Bestellung an das Blisterzentrum per Knopfdruck übermitteln. Im System wird automatisch eine Datei im angeforderten Dateiformat erstellt und versendet. Im Fall von nicht-vorrätigen Medikamenten im Blisterzentrum wird über die Plattform MediMan eine Rückmeldung an die Apotheke gesendet. Die Apotheke kann daraufhin auf Basis des Rahmenvertrages nach § 129 Abs. 2 SGB V und unter Beachtung der dort vorgesehenen Auswahlkriterien ein alternatives Medikament auswählen. Sollte dieses ebenfalls nicht beim Blisterzentrum vorrätig oder ein Austausch nicht möglich sein, muss mit dem Arzt Rücksprache gehalten und bei Bedarf ein neues Rezept angefordert werden. Eine graphische Darstellung des Datenflusses bezogen auf den Auftragseingang zur maschinellen Verblisterung über die Vernetzungsplattform MediMan lässt sich Abb. 5 entnehmen. In der vorliegenden Fallstudie wird die Schnittstelle zur Blistersoftware blister.connect noch eingerichtet. Bis dahin wird das im richtigen Dateiformat erzeugte Dokument bei MediMan heruntergeladen und anschließend bei blister.connect hochgeladen, wo auch die Prüfung der Verfügbar- und Blisterbarkeit erfolgt. Anschließend folgen im Blisterzentrum die Fertigstellung der Blister und die Auslieferung an die Apotheke. Diese überprüft als Arzneimittelexperte die Blister auf Richtigkeit und Qualität, da sie in der Verantwortung steht und die letzte Instanz ist, die Medikamente ausgeben darf. Nach erfolgter Prüfung liefert die Apotheke die verblisterten Medikationen an den Pflegedienst aus.

Die Blister ermöglichen dem Pflegedienst somit eine erleichterte und sichere Vergabe der Medikation an Patienten, bei der die mit dem richtigen Zeitpunkt versehene Verpackung nur geöffnet und der Inhalt verabreicht werden muss, welches Fehler bei der Zusammenstellung der Medikation minimiert. Insgesamt ergibt sich somit das Potenzial für den Pflegedienst, Zeit einzusparen, die in die Versorgung der Pflegebedürftigen investiert werden kann. Dies ist angesichts des Fachkräftemangels in der Branche ein wichtiger Nutzen. Als Vorteile für die Apotheke selbst können die folgenden, zentralen Aspekte von Vernetzungsplattformen genannt werden: Asynchrone Kommunikation, digitales Termin- und Aufgabenmanagement, Sichtbarkeit, Dokumentation, einheitliche Datenbasis sowie Kundenbindung und damit eine stärkere Wettbewerbsposition. Dem gegenüber stehen einmalige Investitionskosten für die Umstrukturierung sowie laufende Lizenzgebühren und Personalressourcen für die Plattformnutzung. Eine Übersicht der Vor- und Nachteile der maschinellen Verblisterung, gesteuert über die Vernetzungsplattform MediMan, lässt sich Tab. 2 in Form einer Argumentebilanz entnehmen.

**Tab. 2:** Argumentebilanz mit Vor- und Nachteilen der maschinellen Verblisterung gesteuert über MediMan

Vorteile	Nachteile
Integration diverser Insellösungen	Manueller Upload des Rezeptes
Bestellung an das Blisterzentrum per Knopfdruck	Bisher keine Integration des Warenwirtschaftssystems
Automatische Erstellung und Versendung der Bestelldatei	Herstellung einer Schnittstelle zur Blistersoftware ist mit Aufwand verbunden
Arbeitserleichterung des Pflegedienstes durch einfache und sichere Vergabe der Medikation	Investitionskosten für die Umstrukturierung
Fehlerreduktion bei der Zusammenstellung der Medikation durch maschinelle Fertigung	Lizenzgebühren
Zeiteinsparung des Pflegedienstes, welches in die Versorgung von Patienten investiert werden kann	Personalressourcen für die Plattformnutzung
Asynchrone Kommunikation	–
Digitales Termin- und Aufgabenmanagement	–
Erhöhte Sichtbarkeit	–
Digitale Dokumentation	–
Einheitliche Datenbasis	–
Stärkung der Kundenbindung bei Apotheken	–
Stärkere Wettbewerbsposition für Apotheken	–

## Diskussion und Implikationen

Durch die Ergebnisse dieser Fallstudie lassen sich die folgenden Rückschlüsse ziehen: Eine maschinelle Verblisterung hat das Potenzial, zu einer Arbeitserleichterung für Pflegedienste oder pflegende Angehörige mit einer reduzierten Fehlerquote bei der Zusammenstellung und Verabreichung der Medikamente beizutragen, wodurch eine höhere Arzneimitteltherapiesicherheit ermöglicht werden könnte. Diese Fehlerreduktion lässt sich durch die vereinfachte Vergabe der Medikamente durch die Pflegekräfte herbeiführen, da der Prozess der Medikationsvergabe durch die vorgefertigten Blister deutlich im Umfang und der Komplexität reduziert wird. Bei der Einbindung von Blisterzentren wird eine Erleichterung im Arbeitsalltag der Apotheken erreicht, sobald die Prozessumstellung nach ggf. anfänglichen Anpassungshürden erfolgt ist. Insgesamt könnte hierdurch nicht nur die Arbeitsbelastung des Personals gesenkt, sondern auch mehr Kapazitäten für die Betreuung der Pflegebedürftigen geschaffen werden. Besonders die COVID-19-Pandemie hat die Potenziale der maschinellen Verblisterung greifbar gemacht. Ein Beispiel stellt der besonders hohe Betreuungsaufwand für multimorbide Chroniker in der Pandemie dar. Es geht darum, besonders schützenswerte Risikopatienten mit höchster Sicherheit und Qualität zu versorgen und unnötige Kontakte zu vermeiden (z. B. durch Reduzierung von (Leer‑)Fahrten), was hierdurch ermöglicht wird. Dennoch führt die maschinelle Verblisterung insgesamt zu einem hohen logistischen Aufwand, da Medikationsänderungen Anpassungen der Verblisterung hervorrufen. An diesem Punkt setzt der hier vorgestellte Anwendungsfall an und stellt dar, wie Vernetzungsplattformen dazu beitragen können, die Logistik, die Kommunikation, das Aufgabenmanagement sowie die Dokumentation aber vor allem auch die Versorgungsqualität erheblich zu vereinfachen und zu verbessern. Dadurch entstehen Möglichkeiten, die weg von mehreren Insellösungen bis hin zu einer einheitlichen, über die Apotheke gesteuerten Plattform gehen und so mehrere Akteure mit der gleichen Datenbasis vernetzt werden können.

Besonders in den ersten vier Wochen nach der Abstimmung mit allen beteiligten Akteuren war der Aufwand hoch. Dies lag nicht, wie erwartet, an der Prozessumstrukturierung, da sich diese aufgrund der hohen Bereitschaft der Mitarbeitenden einfach gestaltete. Stattdessen war es sehr aufwändig, die Schnittstelle zwischen MediMan und der Software des Blisterzentrums blister.connect zu schaffen, obwohl alle technischen Voraussetzungen bei MediMan geschaffen waren. Die Komplexität lag darin, die Blisterdatei in das richtige Format zu bringen, indem die Informationen aus den einzugebenden Feldern in MediMan automatisch zu einem von blister.connect gewünschten Dateiformat zusammengesetzt werden. Nur mit dem richtigen Dateiformat, das sonst aus der Blistersoftware automatisch erstellt wird, kann ein Blisterschlauch automatisch erstellt werden. Um das zu ermöglichen, musste der Blistersoftware-Hersteller die Anforderungen an MediMan teilen, die erst einmal zu bestimmen waren, da es dafür noch keine zentralen Informationen und standardisierten Prozesse gab. Dies beschreibt eine Herausforderung, die für Lösungen im frühen Markt, d. h. in diesem Falle als erste Schnittstelle für den Blistersoftware-Hersteller, typisch sind. Der Blistersoftware-Hersteller, einer der führenden in Deutschland, musste sich den eigenen technischen Voraussetzungen erst bewusst werden, sodass eine Schnittstelle eingerichtet werden konnte. Für weitere Schnittstellen und für alle nachfolgenden Lösungen ist der Weg jedoch geebnet.

Die maschinelle Verblisterung ist aktuell trotz des großen Potenzials noch nicht stark verbreitet. Die Bezahlung von Pflegediensten an die Apotheke wird häufig noch als ein Hinderungsgrund der flächendeckenden Einführung angesehen. Diese belaufen sich nach Aussage von im Vorfeld dieser Fallstudie befragten drei Apothekern bei maschinell gefertigten Schlauchblistern auf etwa fünf Euro pro Woche pro Patient. Für die manuelle Verblisterung berechnen Apotheken meistens zwischen 2–3,50 € pro Woche pro Patient, hinzugerechnet wird noch das Rezept- und Medikationsmanagement (IWW 2021). Hinzu kommt, dass noch keine einheitliche Regelung für die Honorierung von Apotheken für das Verblistern existiert (Müller-Bohn 2014). Darüber hinaus ist die Vergütung für die Unterstützung einer Apotheke für das Verblistern aktuell noch kaum kostendeckend, da ein recht hoher Personal- und Organisationsaufwand für die manuelle wie auch die maschinelle Verblisterung von Medikamenten anfällt (Müller und Schabbeck 2019). Durch den Einsatz maschineller Verblisterung über Vernetzungsplattformen kann dieser Prozess jedoch standardisiert und erleichtert werden. Dennoch ist in Zukunft zu diskutieren, die Vergütung der Apotheke zu erhöhen und die Vorteile und Mehrwerte für die Pflege im Rahmen einer qualitativ hochwertigen Verblisterung entsprechend zu nutzen (IWW 2021). Die Herausforderung besteht dabei darin, dass bislang Pflegedienste bzw. Patienten selbst die Apotheken für die Verblisterung vergüten müssen. Diese Akteure können die Vergütung jedoch nicht erhöhen, um sich das maschinelle Verblisterungsangebot mit Einbindung von Blisterzentren für die Apotheken attraktiver zu gestalten. Ein Ziel könnte es demzufolge sein, dass die Verblisterung als eine Regelleistung der Krankenkassen angeboten wird und die Apotheken dafür entsprechend honoriert werden. Für den Pflegedienst liegen die Vorteile der maschinellen Verblisterung über Vernetzungsplattformen in der Zeitersparnis und der Gewährleistung der Arzneimittelsicherheit durch die Apotheke (Ditzel 2016). In der Arzneimitteltherapie könnten sich darüber hinaus Einsparpotentiale aus vermeidbaren Verschreibungsfehlern von 8,7 Mio. € pro Jahr ergeben. Aus vermeidbaren Über- und Unterdosierungen der Patienten könnten potenziell 1,7 Mio. € pro Jahr eingespart werden. Durch Blister ergebe sich auf ein Jahr gerechnet ein Einsparvolumen von 18,36 € pro Patient (Lauterbach et al. 2006). Insgesamt können aus der Praxisfallstudie somit die folgenden acht Erkenntnisse bzw. Handlungsbereiche festgehalten werden:

1. *Prozessvereinfachung*: Vereinfachung des Prozesses durch eine zentrale Vernetzungsplattform wie MediMan, wodurch ein „veraltetes“ Versorgungsmodell mithilfe von Technologie neu aufgesetzt wird. Auf Basis der Erkenntnisse dieser Fallstudie sollte diese Plattform Pflegedienste, Apotheken, Blisterzentren und Ärzte miteinander vernetzen und einen Datenaustausch hinsichtlich verordneter Rezepte und Medikationspläne erlauben. Die Plattform sollte so übersichtlich und intuitiv gestaltet sein, dass für die Akteure möglichst wenig Aufwand und kaum Abstimmung nötig sind. Zudem sollten standardisierte Prozesse verfolgt werden, die insbesondere für Pflegedienste, ohne Warteschleifen und Zeitverzug auskommen und so mehr Zeit für die Versorgung der Patienten lassen.
2. *Plattformerprobung*: Bestehende Plattformen wie z. B. die Gesundheitsplattform gesund.de^3^, über die u. a. Apotheken gesucht, E‑Rezepte hochgeladen und Medikationslisten angelegt werden können, sollten von den Gesundheitsdienstleistern erprobt werden, um auf zukünftige digitale Lösungen wie das E‑Rezept besser vorbereitet zu sein.
3. *Abstimmungsbedarfe*: Anfangs kann es zu einem höheren Abstimmungs- und Anpassungsbedarf (Prozesse und IT bzw. Business-IT-Alignment) als erwartet kommen, weshalb empfohlen wird, diese frühzeitig und mit vergleichsweise hohem Zeitanteil einzuplanen. Diese Abstimmungsbedarfe zahlen sich mittel- und langfristig aus, sobald die Vernetzungsplattform und der neue Versorgungsprozess standardisiert integriert sind.
4. *Mitarbeitereinbindung und -schulung*: Die frühzeitige Einbindung von Mitarbeitenden und deren Schulung ist von hoher Relevanz, da diese mit der Plattform arbeiten. Es ergeben sich daraus Vorteile wie die Entlastung des Pflegepersonals und eine Erhöhung der Behandlungsqualität sowie die Fehlervermeidung durch standardisierte Kommunikation.
5. *Informationsverbreitung*: Aktuell ist insbesondere die Informationsverbreitung wichtig, damit die Gesundheitsakteure und die Bürger besser über die digitalen Angebote und deren Möglichkeiten informiert werden und sich die maschinelle Verblisterung als Aufgabe der Apotheke vor Ort durchsetzen kann.
6. *Vergütungsregelungen*: Es bedarf an klaren, einheitlichen Vergütungsregelungen durch die Politik, die aktuell noch nicht bestehen.
7. *Schnittstelle Warenwirtschaftssystem*: Für zukunftsfähige Plattformen ist eine Schnittstelle zu bestehenden Warenwirtschaftssystemen von Bedeutung. Die Schnittstelle sollte dabei die Daten beider Systeme abspeichern, sodass der händische Datenabgleich entfällt. Informationen über die Medikamente und deren Stückzahl sollen dabei an die Plattform übermittelt werden. Die Plattform übermittelt dann die Daten der Bestellung an das Warenwirtschaftssystem. Durch die Schnittstelle entfällt die doppelte Pflege beider Systeme und kann somit potenziell zu Zeitersparnissen führen und Erfassungsfehler reduzieren.
8. *Interoperabilität und TI*: Vernetzungsplattformen sollten zudem über offene Schnittstellen und eine entsprechende Kompatibilität zur TI verfügen. Die Vorgaben zur Interoperabilität an Schnittstellen von Plattformen sind für einen sichereren Austausch medizinischer Daten zu beachten, indem die vorhandenen international anerkannten technischen, syntaktischen und semantischen Standards (z. B. HL7 CDA/GDT) berücksichtigt werden.

Ist die Schnittstelle von Apotheke zu Blisterzentrum erstmal geschaffen, ist der Vertrieb der Verblisterung auch niederschwellig an den Patienten möglich. Diese Möglichkeit schafft ein Alleinstellungsmerkmal und damit einen Wettbewerbsvorteil, da der Aufwand für die Realisierung des neuen Versorgungsangebots nur noch sehr gering ist. Um diesen Geschäftszweig erfolgreich zu betreiben, ist es wichtig, alle Mitarbeiter zu schulen, um potenzielle Patienten für die Verblisterung zu gewinnen und pflegende Angehörige auf diese Dienstleistung aufmerksam zu machen. Potenzielle Patienten sind insbesondere die Generation über 60 Jahre sowie Patienten mit Polymedikation und/oder einer schlechten Medikamentencompliance (Ditzel 2016).

Zukünftig ist die Anbindung des pharmazeutischen Großhandels und des zuständigen Blisterzentrums an Vernetzungsplattformen per Schnittstelle wünschenswert, um in Echtzeit die Auftragsabwicklung betreffend Folgebestellungen und die maschinelle Verblisterung zu organisieren. Ziel ist es, einen niederschwelligen und anwenderfreundlichen Prozess abzubilden, mithilfe dessen alle an einer Arzneimittelbeschaffung und am Arzneimittelmanagement beteiligten Akteure durch einen einzigen Arbeitsschritt gleichzeitig informiert werden: Wenn ein Folgerezept für einen Patienten bestellt wird, sollte mit der Auslösung der Bestellung (1) der Großhandel mit der Lieferung des Arzneimittels, (2) die Arztpraxis mit der Ausstellung eines Folgerezeptes und (3) das Blisterzentrum mit der Produktion des Schlauchblisters beauftragt werden. Während des ganzen Ablaufs sind die zuständige Apotheke und der jeweilige Pflegedienst über den Prozessfortschritt gleich informiert und somit können sie direkt und vorab logistische Arbeitsschritte einleiten und Wartezeiten oder Leerfahrten reduziert werden.

Im Folgenden sind einige Limitationen zu nennen, die die Ergebnisse betreffen: Kritische Aspekte umfassen u. a., dass dem Pflegepersonal gegebenenfalls nicht mehr die Kompetenz in Bezug auf Arzneimittel zugeschrieben wird und die maschinelle Verblisterung einiger Arzneimittel bisher ein relativ komplexer Prozess ist (IQWiG 2019). Eine Herausforderung bei der Nutzung von Vernetzungsplattformen besteht auf Seiten der Pflegebedürftigen, die auf Unterstützung bei der Medikation angewiesen sind. Personen, die multimorbid und nicht in der Lage sind, die Medikation selbstständig zu stellen und einzunehmen, könnten durch die eigene Nutzung von Vernetzungsplattformen wie MediMan überfordert sein und Probleme damit haben, diese zu nutzen. Der Mehrwert könnte allerdings im Bereich der pflegenden Angehörigen gesehen werden, die einen besseren Überblick über die Medikation ihres Angehörigen bekommen und somit ein Gefühl der Sicherheit und sicheren Versorgung erhalten könnten. Ein weiterer Kritikpunkt besteht bei den Änderungen der Medikation durch Ärzte. Diese Änderungen können durch manuelles Stellen der Medikation unmittelbar durch den Pflegedienst umgesetzt werden. Bei der maschinellen Verblisterung ist es deshalb besonders relevant, dass alle Stakeholder Vernetzungsplattformen kontinuierlich nutzen, um den relevanten Personen etwaige Änderungen schnellstmöglich zu übermitteln und Medikationen anzupassen. Eine weitere methodische Limitation liegt darin, dass es sich hier um eine Fallstudie handelt, sodass eine empirische Untersuchung nicht ohne Weiteres möglich ist und die Ergebnisse, die sich hier ergeben, nicht notwendigerweise auf andere Bereiche übertragbar und generalisierbar sind.

Insgesamt besteht aufgrund der fehlenden Datengrundlage im Bereich Verblisterung ein großer Bedarf an Studien vor allem in Deutschland, um die Nutzen- und Kosten-Aspekte in der Patientenversorgung durch den Einsatz der patientenindividuellen Verblisterung zu untersuchen (IQWiG 2019). Aus diesem Grund wird hier ermutigt, eine Kosten-Nutzen-Analyse im Bereich der maschinellen Verblisterung anhand von Vernetzungsplattformen durchzuführen.

## Fazit

Ziel dieses Beitrages war es, aufzuzeigen, welche Potenziale sich aus der maschinellen Medikamentenverblisterung kombiniert mit einer Vernetzungsplattform wie MediMan ergeben, um die Zusammenarbeit zwischen Apothekern und Pflegediensten zu verbessern (FF1) sowie Erkenntnisse und Handlungsbereiche auf Basis dieser Fallstudie in Bezug auf die maschinelle Verblisterung gesteuert über die Vernetzungsplattform MediMan abzuleiten (FF2). Mit Rückschluss auf FF1 lässt sich sagen, dass sich Potenziale im Bereich der Prozesskosten für Apotheken und Pflegedienste ergeben könnten, welche sich langfristig senken lassen könnten. Hierdurch entstünde Potenzial, um die eingesparten Ressourcen in die Behandlungsqualität zu investieren und die Behandlungsqualität somit zu steigern. Zudem könnten Medikationsfehler über die maschinelle Verblisterung und den hier angegebenen Prozess reduziert werden. Darüber hinaus kann durch die Einbindung von Blisterzentren der Arbeitsaufwand für Apotheken weiter verringert und die Arzneimittelsicherheit für Pflegedienste gewährleistet werden. Der hier vorgestellte Zielprozess zeigt einen Schritt weg von Insellösungen und zeigt das Potenzial hin zu einer einheitlichen Lösung für alle Akteure auf, die an der medikamentösen Versorgung eines Patienten beteiligt sind. Hieraus entsteht das Potenzial, Apotheken, Pflegedienste und auch Arztpraxen über Institutionsgrenzen hinweg digital zu vernetzen und somit die Zusammenarbeit nachhaltig zu verbessern. Mit Blick auf FF2 lässt sich festhalten, dass sich anhand dieser Fallstudie acht Erkenntnisse bzw. Handlungsbereiche ableiten lassen: (1) Prozessvereinfachung, (2) Plattformerprobung, (3) Abstimmungsbedarfe, (4) Mitarbeitereinbindung- und schulung, (5) Informationsverbreitung, (6) Vergütungsregelungen, (7) Schnittstelle Warenwirtschaftssystem sowie (8) Interoperabilität und TI. Offene Fragen beziehen sich auf Kosten-Nutzen-Aspekte der maschinellen Verblisterung, welche über Vernetzungsplattformen gesteuert werden und auf noch ungeregelte Vergütungsregelungen für die maschinelle Verblisterung, welche in zukünftiger Forschung adressiert und ausgearbeitet werden sollten, um den Ausbau maschineller Verblisterung, gesteuert über Vernetzungsplattformen, voranzutreiben.

## Footnotes

1 Pharmatechnik. Lösungen Verblisterung: https://www.pharmatechnik.de/loesungen/vernetzung/verblisterung.

2 MediMan. Medikationsmanagement für Apotheken: https://www.mediman.de/start.

3 https://www.gesund.de/.
